# Clinical study on the cerebral infarction accompanied with septic disseminated intravascular coagulation

**DOI:** 10.20407/fmj.2020-011

**Published:** 2020-11-13

**Authors:** Tetsuharu Kako, Fumika Azuma, Kazuya Nokura, Hideo Izawa

**Affiliations:** 1 Department of Neurology, Fujita Health University Bantane Hospital, Nagoya, Aichi, Japan; 2 Department of Cardiology, Fujita Health University Bantane Hospital, Nagoya, Aichi, Japan

**Keywords:** Sepsis, Disseminated intravascular coagulation, Cerebral infarction, Body temperature, Blood pressure

## Abstract

**Objectives:**

Patients with disseminated intravascular coagulation (DIC) due to sepsis often develop cerebral infarction; but the frequency, mechanism of onset and prognosis have not been fully elucidated. We reported courses and characteristics of septic DIC cases hospitalized in our hospital in the present study.

**Methods:**

Patients with septic DIC who underwent brain imaging were selected. Vital signs, disorders of consciousness and blood test results at the time of onset were compared between cases that developed cerebral infarction (cerebral infarction group) and those that did not (non-infarction group).In cases of cerebral infarction, the site and the size of the infarct lesion were also described.

**Results:**

In 27 septic DIC patients who underwent brain imaging, eight patients had cerebral infarction. Although the percentage of patients who survived in the cerebral infarction group (2/8, 25%) was lower than that in the non-infarction group (7/17, 37%), , no significant difference was observed as both group showed poor prognoses. Those two patients who survived in the cerebral infarction group had severe consciousness disturbance and poor functional prognosis. Although the body temperature was significantly lower and the blood pressure was higher in the cerebral infarction group, no significant difference was found in general blood tests, so we thought it would be necessary to look for other markers that could be indicators for the risk of cerebral infarction.

In the cerebral infarction group, two cases had a single lesion, and six cases had multiple lesions. Of the latter, two cases had massive lesions with a diameter of 1.5 cm or greater, four cases had only small lesions with a diameter of less than 1.5 cm, and two cases had a mixture of both. Most of the patients had lesions in the vertebrobasilar artery, which suggested that the pathogenesis involves not only embolism due to microthrombi, but also vasculitis and intravascular inflammation.

**Conclusions:**

Cerebral infarction was observed highly frequently; eight out of 27 cases (29.6%) when brain imaging was undergone in septic DIC patients. The prognosis of patients with cerebral infarction was poor, but no difference from the non-infarction group was observed. In addition to embolism, the presence of inflammation is considered to be important for the onset. In order to predict the prognosis and determine a suitable treatment, it would be recommended to undergo brain imaging when patients with septic DIC have consciousness disturbance or elevated blood pressure, and do not have fever.

## Introduction

Disseminated intravascular coagulation (DIC) is a condition in which microthrombi are formed in a blood vessel due to infection or a cancer. In recent years, the relationship with microcirculatory disturbance has been emphasized, and the condition is now defined as enhancement of intravascular coagulation that can cause organ damage.^1^ It is thought that damage to vascular endothelial cells and the progression of microcirculatory disturbance lead to multiple organ failure. Microthrombi are mainly found in the kidney and lungs, and they may cause renal failure and acute lung injury (ALI).^2^

In 1865, Trousseau reported that systemic embolism (particularly cerebral embolism) due to a cancer as phlebitis and venous thrombosis,^3^ and subsequently, the concept was established as multiple thrombosis due mainly to DIC caused by adenocarcinoma and endocarditis. Recently, a few studies on cerebral infarction associated with infectious diseases have been conducted,^4^ and it has been reported that about 25% of patients with infective endocarditis have neurological complications.^5^

On the other hand, some patients who consult the neurology department with chief complaints of paralysis and consciousness disturbance may develop cerebral infarction due to septic DIC, but there have been very few studies of frequency and prognosis.

In this study, we investigated the background and prognosis of patients with cerebral infarction complicated by infectious DIC, and examined whether there was a difference from patients who did not have cerebral infarction.

## Methods

Our hospital is a general hospital with 453 beds. From patients admitted to our hospital during the three years from 2011 to 2013, patients with a diagnosis of DIC were selected. Those in whom the underlying disease was confirmed to be an infectious disease were selected, while patients with cancers and those in whom an underlying infectious disease could not be identified were excluded. Further, using the acute DIC score^6^ developed by the Japanese Association for Acute Medicine and the Japanese Society on Thrombosis and Hemostasis, those with a score of 4 or higher were diagnosed as DIC. Cases with a DIC score of 3 or less were excluded. Patients who underwent CT or MRI when DIC was diagnosed were the final target. For these patients, using the SOFA score^7^ which is the diagnostic criteria for sepsis; if there was an increase of 2 or more, these patients were confirmed to have sepsis.

Among the selected septic DIC patients, vital signs (blood pressure, body temperature, pulse rate, respiratory rate) at the onset, presence/absence of consciousness disturbance, and blood test results (blood count, biochemistry, coagulation test) were retrospectively investigated. Diagnosis of cerebral infarction was made by the appearance of a low density area on CT, or the appearance of a high signal on a MR diffusion-weighted image. Using these data, we examined whether there was a difference between the group with cerebral infarction and the non-cerebral infarction group.

## Statistical analysis

EzR^8^ and Excel were used as statistical packages. In order to examine the sex difference in the occurrence of cerebral infarction, the absence/presence of sepsis and the survival rate, the patients were first divided into two groups according to whether or not they had cerebral infarction. Then, the number of cases was recorded respectively according to the sex, presence/absence of sepsis and survival/death, and the data was tested using the χ² test. For comparison of age, blood test data and vital signs, the Wilcoxon rank sum test was used to examine the numerical values of each item in the two groups, i.e., eight cases with cerebral infarction and 19 cases without cerebral infarction. For all the items, p<0.05 was considered as statistically significant.

## Ethical considerations

This study was conducted with the approval of the Fujita Medical University Ethics Committee (CI18-273). Great care was taken to protect patient privacy. Since this was a retrospective study, the number of patients was large: Hence, informed consent was not possible to be obtained, and opt-out was performed.

## Results

### Selection of study cases

**Figure 1:**
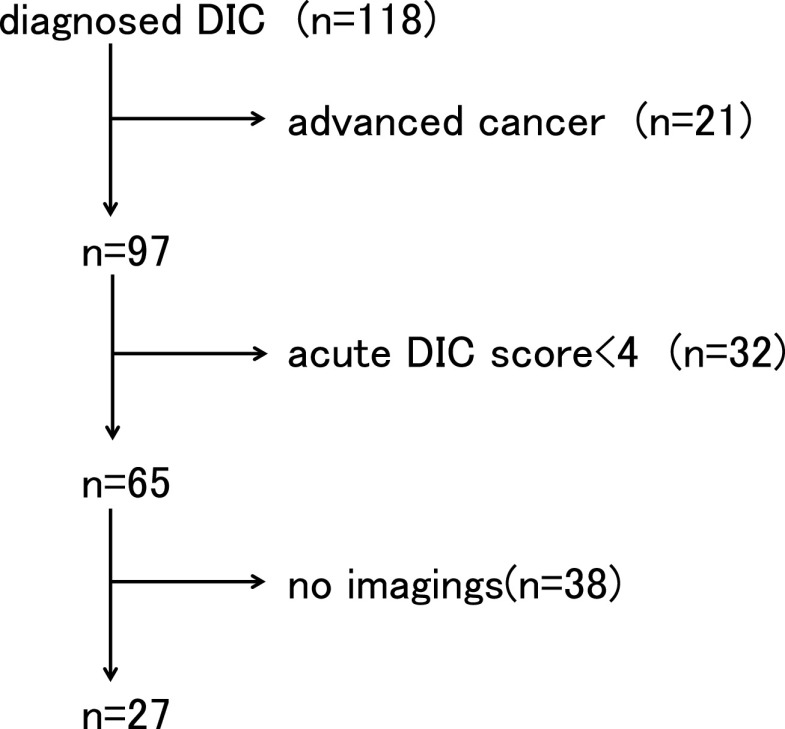
The exclusion diagnosis algorithm.

The number of patients admitted to our hospital between 2011 and 2013 was 25017, of which 118 (0.47%) were registered with DIC as the name of the disease. Of these, 21 cases were DIC patients with malignant tumor as the underlying disease, and there were 97 DIC cases excluding non-infectious diseases. Next, 32 patients with an acute DIC score of 3 or less were excluded, and the remaining 65 patients were diagnosed with DIC. Of these, 51 cases had consciousness disturbance at the time of DIC diagnosis, and in 27 cases, head CT or MRI was performed after DIC developed and consciousness disturbance appeared (Figure 1). All of these 27 cases met the diagnostic criteria for sepsis using the SOFA score. Of the 27 patients who underwent brain imaging, the onset of cerebral infarction was confirmed in eight cases (29.6%).

### Examination of 27 cases for which brain imaging diagnoses were performed

A comparative study was undertaken by dividing the 27 cases undergoing brain imaging into two groups; eight cases with cerebral infarction, and 19 cases without cerebral infarction.

Table 1 shows the background such as age and underlying disease. The average age of the cerebral infarction group was 84.87±9.16 years, and the average age of the non-infarction group was 76.10±16.18 years; and there was no significant difference (p=0.10). There were 14 males and 13 females, and cerebral infarction was observed in four of each; thus there was no gender difference as to the occurrence of cerebral infarction (respectively, p=0.90). Out of 27 cases, nine (33.3%) survived. Regarding survival rates, the prognosis was poor in both the cerebral infarction and the non-infarction groups, but no significant difference was observed (p=0.21). Severe disturbance of consciousness remained in the two survivors of the cerebral infarction group, and the functional prognosis was also poor.

**Figure 2:**
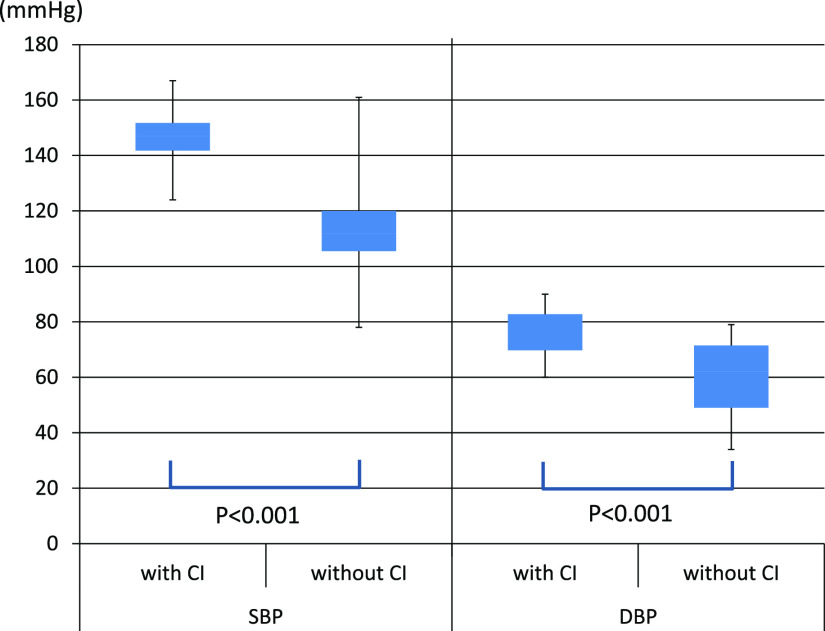
Comparison of the blood pressures of the cerebral infarction group and the non-infarction group. Both the systolic and diastolic blood pressures were significantly increased in the cerebral infarction group. SBP=Systolic blood pressure, DBP=Diastolic blood pressure, CI=Cerebral infarction.

**Figure 3:**
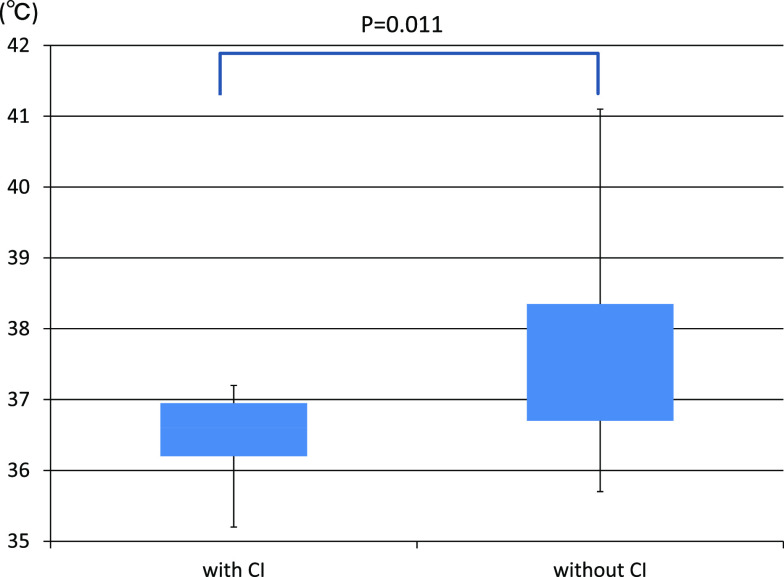
Comparison of the body temperature of the cerebral infarction group and the non-infarction group. The cerebral infarction group had lower body temperature.

Next, vital signs and blood test results were compared between the cerebral infarction group and the non-infarction group (Table 2). In terms of vital signs, both systolic blood pressure and diastolic blood pressure were significantly higher in the cerebral infarction group (p<0.001) (Figure 2). The pulse rate tended to be lower in the cerebral infarction group, but there was no significant difference (p=0.11). In the cerebral infarction group, no increase in body temperature was observed, and all cases were 37.5°C or lower, whereas in the non-infarction group higher values were frequently observed, and a significant difference was found (p=0.011) (Figure 3).

Blood test results showed that FDP were higher than 25 μg/mL, which is three points in the acute DIC score, in all eight cases of the cerebral infarction group, but no significant difference was observed compared with the non-infarction group (p=0.99). Regarding the platelet count, the mean value was 93000/μL in the cerebral infarction group and 79000/μL in the non-infarction group, but there was also no significant difference (p=0.96). The mean CRP value for the inflammatory response was 16.70 mg/dL in the infarct group and 12.53 mg/d in the non-infarct group, which tended to be higher in the infarct group, but there was no significant difference (p=0.16). PT-INR was slightly longer in the non-infarction group than in the cerebral infarction group, but again no significant difference was observed (p=0.08).

### Image findings of cerebral infarction cases

**Figure 4:**
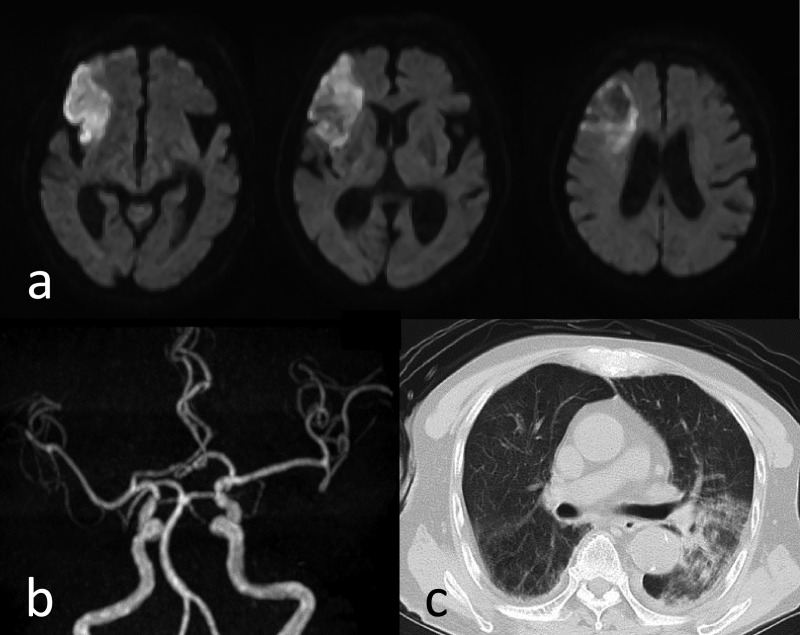
Case 8: 89-year-old male. Cerebral infarction was a single lesion, and it was massive (over 15 mm). a Brain MR DWI showed cerebral infarction in the anterior half of the right middle cerebral artery region. b MRA indicated the possibility that a branch of the middle cerebral artery was occluded. c Chest CT showed pneumonia.

**Figure 5:**
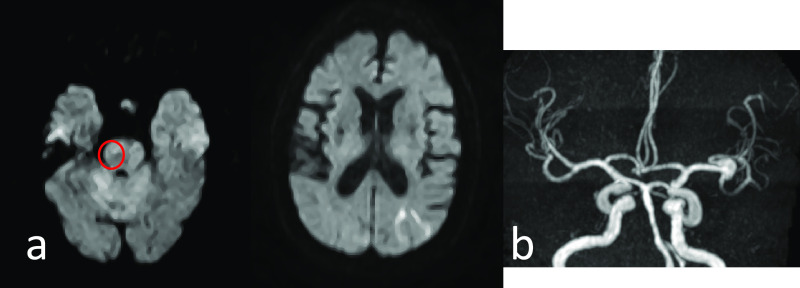
Case 4: 86 year old male. A case with multiple small infarctions. He had marked consciousness disturbance, and required emergency hospital admission. a MRI showed acute cerebral infarction of less than 15 mm in the middle part of the right pons and the left occipital region. b MRA showed no obvious abnormalities.

**Figure 6:**
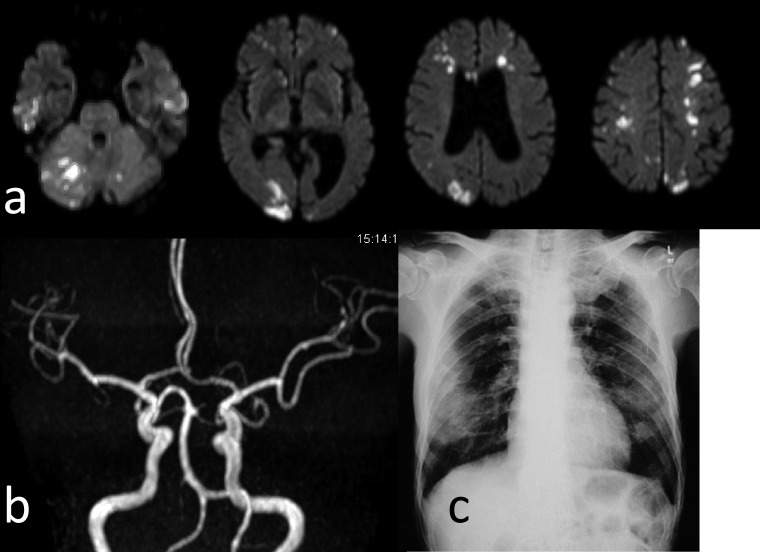
Case 1: 72-year-old male. He required emergency transport because of difficulty walking. This was a mixed case of a massive lesion+small lesions. a Brain MRI showed a lesion of greater than 15 mm+multiple small infarctions. b MRA did not show disruption of the main artery. c Chest X-ray showed an image of pneumonia.

The characteristics of the lesions in the eight patients with cerebral infarction were examined (Table 3). There were two cases with a single lesion, and six cases with multiple lesions. Of these, two cases had massive lesions with a diameter of 1.5 cm or more (Figure 4), four cases had only small lesions with a diameter of less than 1.5 cm (Figure 5), and two cases had a mixture of both (Figure 6). Six of the eight cases had a marked consciousness disturbance with a GCS of six or less at diagnosis. In the eight cerebral infarction cases, infarction was also observed in the vertebrobasilar artery system in six cases, and there was also one case where cerebral infarction was not found in the internal carotid artery system (middle cerebral artery region, etc.). In the six cases with multiple lesions, head MRA showed no occlusion of the main artery.

## Discussion

The essential pathogenesis of DIC is a combination of thrombosis and inflammatory response, which can be divided into two types, a fibrinolysis-promoting type and fibrinolysis-inhibiting type. In DIC due to inflammation, the latter type is more common, and necessarily causes ischemic lesions. Upon invasion by pathogens, dendritic cells and macrophages inside epithelial cells recognize them as pathogen molecules (collectively referred to as PAMPs (Pathogen-Associated Molecular Patterns), such as endotoxins), resulting in the generation of cytokines and HMGB1 (high mobility group box-1 protein). HMGB1 and cytokines induce tissue repair, but if PAMPs are excessive and spread throughout the body, they cause DIC. Hatada et al. have demonstrated that the simultaneous presence in blood vessels of HMGB1 and thrombin, which are substances related to this inflammation, causes DIC.^9^ Moreover, in sepsis, the production of thrombomodulin and t-PA is reduced by inflammatory cytokines, and fibrinolysis is inhibited,^10^ which leads to thrombus formation. There is also a study stating that in addition to these reactions, in brain tissue, thromboplastin is high and the amount of thrombomodulin is low, making it prone to embolism.^11^ At the same time, although thrombomodulin is found in the brain, it is less distributed in the pons and putamen, which are common sites of cerebral infarction, and there is a view that thrombomodulin works to suppress the formation of infarction also in the brain.^12^ Septic DIC is considered to be the fibrinolysis-inhibited form of DIC, in which cases with high plasminogen activator inhibitor-1 (PAI-1) levels are associated with poor prognosis and are more likely to result in organ damage caused by circulatory disturbances.^13^

The laboratory tests measured during the course of DIC in this study varied from case to case. As described above, markers associated with organ symptoms in fibrinolysis-inhibited DIC include PAI-1, antithrombin III (AT-III), protein C, HMGB-1, leukocyte elastase fractionated fibrin degradation products (e-XDP) and soluble fibrin (SF), but they were measured in only a few cases. In this study, we found no significant differences in common parameters such as platelet count, FDP, and C-reactive protein (CRP), which are indicators of DIC status, between the infarct group and non-infarct group. Hence, it is necessary to look for other markers that predict the onset of cerebral infarction. In future, it will also be necessary to collect a large number of cases with a wider range of test items, and to undertake a prospective study.

In six out of eight cases of cerebral infarction complicated by septic DIC, cerebral infarction was also observed in the vertebrobasilar artery system, and conversely, there was also a case with no cerebral infarction in the internal carotid artery system. In collagen disease (systemic lupus erythematosus), it has been reported that vasculitis is involved in the onset of cerebral infarction, and vertebrobasilar artery lesions were found in many cases.^14^ Based on these findings, it is considered that the pathogenesis of cerebral infarction of infectious DIC is due not only to microthrombi floating in the blood vessels, but also vasculitis and inflammatory reactions in the blood vessels. There are also cases of infarction in the internal carotid system that are indistinguishable from ordinary atherothrombotic cerebral infarction (Figure 4), and atherosclerosis may indeed be a risk factor for cerebral infarction triggered by DIC.

The causative disease of DIC was pneumonia in five of the eight patients (Table 3). Microemboli via the general circulation are usually entrapment in the lungs and do not reach the brain. Emboli to the brain often originate from the pulmonary circulation, and if there is inflammation in the lungs, emboli generated in the pulmonary circulation are more likely to cause embolism in the brain. However, our study could not prove the hypothesis that pneumonia is likely to cause cerebral infarction.

In the eight cases with cerebral infarction, the systolic and diastolic blood pressures were significantly higher, and bradycardia tended to occur. When the intracranial pressure increases due to the onset of cerebral infarction or cerebral hemorrhage, the blood pressure is increased by sympathetic nerve stimulation, resulting in bradycardia. This is known as Cushing’s reflex.^15^ Patients with severe infections and sepsis tend to have lower blood pressure due to shock, and the SOFA score and qSOFA score used to predict sepsis^7^ also include decreased systolic blood pressure and the mean arterial pressure as diagnostic criteria. However, blood pressure is significantly increased in the cerebral infarction group, and Cushing’s reflex is considered to occur in septic DIC patients. In septic DIC patients, if their blood pressure is normal or high, it is necessary to consider that they may also be experiencing cerebral infarction.

The mechanism whereby body temperature did not rise in the cerebral infarction group is not clear, but it has been reported that body temperature does not rise even under stress due to damage of orexin-positive cells in the hypothalamus.^16^ Damage of orexin-positive cells in the hypothalamus due to inflammation and microemboli in the blood vessels of the vertebrobasilar arterial system may be involved.

In this study, 51 out of 65 DIC patients had consciousness disturbance, of which 27 (52.9%) underwent brain imaging, which was a low rate. Cerebral infarction was confirmed at a high rate in eight cases (29.6%) among the 27 cases where brain imaging was actually performed by the attending physician, so if the other patients also had had a brain imaging examination, it is possible that the onset of cerebral infarction might have been detected. It has long been considered that DIC causes encephalopathy due to various factors, resulting in consciousness disturbance.^17^ In this paper, DIC encephalopathy includes cerebral infarction, white matter lesions, and PRES (posterior reversible encephalopathy syndrome) observed on head MR. DIC may be treated by a department not specializing in neurological disorders, such as an emergency doctor; and in DIC patients with a consciousness disturbance, head CT or MRI should always be conducted to decide whether or not to administer a neuroprotective drug or prevent cerebral edema. In addition, more than half of the patients with cerebral infarction who suffered from septic DIC were admitted to the neurology department (Table 3). However, since treatment of the underlying disease is the most important factor in the DIC state, and the neurologist must collaborate with a specialist in the infected organ.

In the treatment of DIC, it is recommended to administer AT-III preparations as anticoagulant therapy when antithrombin activity is decreased.^18^ In many cases of DIC associated with infectious disease, AT-III activity decreases, so administration of AT preparations is often necessary. Because the concept of DIC is not widely known abroad, their effectiveness has not been proven.^19^ But in Japan, in a clinical study on DIC cases from around 2009, it was reported that the recovery rate from DIC was significantly high.^20^ Also in Japan, although thrombomodulin preparations can be used for the treatment of DIC, they were not adopted in the Japanese Clinical Practice Guidelines for Management of Sepsis and Septic Shock 2016. However, studies are still ongoing, and they are reported to be effective in some patients.^21^ It has also been reported that cytokine adsorption by blood purification therapy is effective for the treatment of sepsis,^22^ and if these treatment interventions allow early recovery from septic DIC, they may lead to inhibition of the onset of cerebral infarction, which is a future goal.

## Supplementary Material

PDF-Japanese

## Figures and Tables

**Table1:** Age, gender, and underlying diseases of 27 patients with septic disseminated intravascular coagulation according the occurrence of cerebral infarction.

	Cerebral infarction (+)	Cerebral infarction (−)
Gender(Male/Female)	4/4	12/7
Age	84.87±9.16	76.1±16.18
Number of survivors	2	7
Underlying diseases		
Pneumonia	5	13
Urinary-tract infection	2	0
Others	1	6

**Table2:** Mean value of body tempereture, blood pressure, pulse rate, and levels of inflammatory and coagulation markers in septic disseminated intravascular coagulation patients with or without cerebral infarction.

		Cerebral infarction (+) n=8	Cerebral infarction (−) n=19	p value
Body tempereture	°C	36.55±0.48	37.57±1.13	0.011
Systolic blood pressure	mmHg	146.13±13.33	113.25±19.19	<0.001
Diastolic blood pressure	mmHg	74.88±9.25	60±13.03	<0.001
Pulse rate	/min	92.62±20.62	106.47±22.40	0.11
White blood cells	/μL	14950±8519.90	14584.90±9919	0.92
Pletelets	×10^4^/μL	10.36±6.97	10.57±8.36	0.96
Fibrin degradation product	μg/mL	123.05±84.34	122.50±206.67	0.99
PT-INR		1.21±0.91	1.91±1.52	0.08
C-reactive protein	mg/dL	16.70±5.97	12.53±7.12	0.16

PT-INR=Prothrombin Time International Normalized Ratio; p value is based on Wilcoxin rank sum test.

**Table3:** Age, gender, type of infection, type of infarction, assessment score, admitted department of 8 cases with cerebral infarction.

No.	Age	Gender	Type of infection	massive/multiple	Area of infarction	DIC score	GCS	Admission
1	72	Male	Pneumonia	massive+multiple	PCA, PICA, WS	4	15	Neurology
2	105	Female	Pneumonia	multiple	PCA, MCA	6	5	Respiratory medicine
3	83	Female	Enteritis	massive	MCA	7	5	Gastroenterology
4	86	Male	Urinary tract infection	multiple	PCA	6	4	Neurology
5	85	Female	Urinary tract infection	multiple	AICA, MCA, WS	4	3	Neurology
6	77	Female	Pneumonia	massive+multiple	PCA, WS	4	3	Respiratory medicine
7	82	Female	Pneumonia	multiple	PICA, PCA, MCA	8	14	Neurology
8	89	Male	Pneumonia	massive	MCA	4	3	Neurology

MCA=Middle cerebral artery, ACA=Anterior cerebral artery, PCA=Posterior cerebral artery, PICA=Posterior inferior cerebellar artery, WS=Watershed of MCA/ACA or PCA, GCS=Glasgow coma scale, DIC=Disseminated intravascular coagulation

## References

[1] Taylor FB, Jr, Toh CH, Hoots WK, Wada H, Levi M. Towards a definition, clinical and laboratory criteria, and a scoring system for disseminated intravascular coagulation. Thromb Haemost 2001; 86: 1327–1330. 11816725

[2] Matsubayashi J, Wakasa T, Yanai H. Thrombosis/embolism and organ dysfunction. Jpn J Diagn Pathol 2018; 35: 264–279 (in Japanese).

[3] Trousseau A. Phlegmasia alba dolens. Clin Med Hotel Dieu De Paris 1865; 3: 94.

[4] Syrjanen J. Infection as a risk factor for cerebral infarction. Eur Heart J 1993; 14 Suppl K: 17–19. 8131782

[5] Sotero FD, Rosario M, Fonseca AC, Ferro JM. Neurological Complications of Infective Endocarditis. Curr Neurol Neurosci Rep 2019; 19: 23. 30927133 10.1007/s11910-019-0935-x

[6] Gando S, Iba T, Eguchi Y, et al. A multicenter, prospective validation of disseminated intravascular coagulation diagnostic criteria for critically ill patients: comparing current criteria. Crit Care Med 2006; 34: 625–631. 16521260 10.1097/01.ccm.0000202209.42491.38

[7] Seymour CW, Liu VX, Iwashyna TJ, Brunkhorst FM, Rea TD, Scherag A, Rubenfeld G, Kahn JM, Shankar-Hari M, Singer M, Deutschman CS, Escobar GJ, Angus DC. Assessment of Clinical Criteria for Sepsis: For the Third International Consensus Definitions for Sepsis and Septic Shock (Sepsis-3). Jama 2016; 315: 762–774. 26903335 10.1001/jama.2016.0288 PMC5433435

[8] Kanda Y. Investigation of the freely available easy-to-use software ‘EZR’ for medical statistics. Bone Marrow Transplant 2013; 48: 452–458. 23208313 10.1038/bmt.2012.244 PMC3590441

[9] Hatada T, Wada H, Nobori T, Okabayashi K, Maruyama K, Abe Y, Uemoto S, Yamada S, Maruyama I. Plasma concentrations and importance of High Mobility Group Box protein in the prognosis of organ failure in patients with disseminated intravascular coagulation. Thromb Haemost 2005; 94: 975–979. 16363239 10.1160/TH05-05-0316

[10] Levi M, van der Poll T, ten Cate H, van Deventer SJ. The cytokine-mediated imbalance between coagulant and anticoagulant mechanisms in sepsis and endotoxaemia. Eur J Clin Invest 1997; 27: 3–9. 9041370 10.1046/j.1365-2362.1997.570614.x

[11] Uchiyama S. Paraneoplastic neurological syndromes: Trousseau syndrome. Nihon Naika Gakkai Zasshi 2008; 97: 1805–1808 (in Japanese). 18833691 10.2169/naika.97.1805

[12] Wong VL, Hofman FM, Ishii H, Fisher M. Regional distribution of thrombomodulin in human brain. Brain Res 1991; 556: 1–5. 1657303 10.1016/0006-8993(91)90540-c

[13] Morishita E. Other molecular markers related to DIC diagnosis and their significances. Journal of Clinical Laboratory Medicine 2018; 62: 1032–1039 (in Japanese).

[14] Ioannidis S, Mavridis M, Mitsias PD. Ischemic stroke as initial manifestation of systemic lupus erythematosus: A case report and review of the literature. eNeurologicalSci 2018; 13: 26–30. 30505957 10.1016/j.ensci.2018.11.001 PMC6251781

[15] Fodstad H, Kelly PJ, Buchfelder M. History of the Cushing reflex. Neurosurgery 2006; 59: 1132–1137; Discussion 7. 10.1227/01.NEU.0000245582.08532.7C 17143247

[16] Zhang W, Sunanaga J, Takahashi Y, Mori T, Sakurai T, Kanmura Y, Kuwaki T. Orexin neurons are indispensable for stress-induced thermogenesis in mice. J Physiol 2010; 588: 4117–4129. 20807795 10.1113/jphysiol.2010.195099 PMC3002445

[17] Siami S, Annane D, Sharshar T. Encephalopathy in sepsis. Crit Care Clin 2008; 24: 67–82, viii. 10.1016/j.ccc.2007.10.001 18241779

[18] Kienast J, Juers M, Wiedermann CJ, Hoffmann JN, Ostermann H, Strauss R, Keinecke HO, Warren BL, Opal SM. Treatment effects of high-dose antithrombin without concomitant heparin in patients with severe sepsis, with or without disseminated intravascular coagulation. J Thromb Haemost 2006; 4: 90–97. 16409457 10.1111/j.1538-7836.2005.01697.x

[19] Warren BL, Eid A, Singer P, et al. Caring for the critically ill patient. High-dose antithrombin III in severe sepsis: a randomized controlled trial. Jama 2001; 286: 1869–1878. 11597289 10.1001/jama.286.15.1869

[20] Gando S, Saitoh D, Ishikura H, et al. A randomized, controlled, multicenter trial of the effects of antithrombin on disseminated intravascular coagulation in patients with sepsis. Crit Care 2013; 17: R297. 24342495 10.1186/cc13163 PMC4057033

[21] Ito T, Thachil J, Asakura H, Levy JH, Iba T. Thrombomodulin in disseminated intravascular coagulation and other critical conditions—a multi-faceted anticoagulant protein with therapeutic potential. Crit Care 2019; 23: 280. 31416465 10.1186/s13054-019-2552-0 PMC6694689

[22] Hirasawa H, Oda S, Nakamura M, Watanabe E, Shiga H, Matsuda K. Continuous hemodiafiltration with a cytokine-adsorbing hemofilter for sepsis. Blood Purif 2012; 34: 164–170. 23095416 10.1159/000342379

