# Supplementary material for: Clinical study on the cerebral infarction accompanied with septic disseminated intravascular coagulation
Source: Fujita Med J. 2020 Nov 13;7(3):99–104. doi: 10.20407/fmj.2020-011 (PMC8749497; doi:10.20407/fmj.2020-011)
Supplement: Supplementary file 1 — PDF-Japanese [file fmj-7-099_s001.pdf]

【要約】

目的

　敗血症により播種性血管内凝固症候群(DIC)を生じた患者では脳梗塞を合併することがあるがその頻度や発症機序、予後について十分な解明がされておらず、当院での入院症例をもとに検討を行った。

方法

　敗血症性 DIC 患者のうち頭部画像検査を施行された患者を抽出し、発症時のバイタルサイン、意識障害の有無、血液検査所見を調査し、それぞれの項目について脳梗塞を発症した群と非発症群の間に差があるか検討した。脳梗塞を認めた症例については発症部位や梗塞巣の大きさも比較検討した。

結果

　敗血症性 DIC 患者の中で頭部画像検査が施行されたのは 27 例、そのうち 8 例に脳梗塞を認めた。脳梗塞発症群と非発症群で比較した結果、脳梗塞発症群の中で生存したのは 2 例で生存率は 25%であったが、非発症群の生存率が 37%でどちらも予後不良であり有意差は認められなかった。脳梗塞発症群で生存した 2 例は重度の意識障害が残存し機能予後も不良であった。脳梗塞発症群では有意に体温が低く、血圧が高値であったが、一般的な血液生化学検査では有意差を認めず、脳梗塞発症の指標となるマーカーのさらなる検索が必要と考えた。

　脳梗塞は 2 例が単発病変、6 例は多発性で径が 1.5cm 以上の massive　2 例、1.5cm 以下の small のみ 4 例、両者の混合は 2 例にみられた。椎骨脳底動脈系に病変を認める患者が多く、発症機序として微小血栓による塞栓症だけでなく血管炎や血管内の炎症の存在が関与していると考えた。

結論

　敗血症性 DIC 患者において頭部画像診断を施行すると 27 例中 8 例(29.6%)と高率に脳梗塞を認めた。脳梗塞を発症した患者の予後は不良であるが非発症群との差は認められなかった。発症には塞栓症の他に炎症の存在が重要であると考えられる。予後予測や治療方針の決定のため、敗血症性 DIC 患者が意識障害や血圧上昇を認め、発熱を認めない場合には頭部画像検査の施行が勧められる。

Key words

Sepsis, DIC, Cerebral infarction, Body temperature, Blood pressure

はじめに

播種性血管内凝固症候群(DIC)は、感染症や悪性腫瘍などにより引き起こされる血管内に微小血栓を形成した状態である。近年は微小循環障害との関連が重要視されており、臓器障害の原因となりうる血管内凝固活性の亢進と定義されている[1]。血管内皮細胞の障害や微小循環障害の進行により多臓器不全を来すと考えられており、微小血栓は主に腎や肺に多く認められ、腎不全や急性肺障害(ALI)の原因となるとされている[2]。

悪性腫瘍により引き起こされる全身性塞栓症(特に脳塞栓症)は1865年にTrousseauによって静脈炎および静脈血栓症として初めて提唱され[3]、のちに主に腺癌によって引き起こされる DIC および心内膜炎から生じる多発性血栓症として概念が確立した。最近では感染症に伴う脳梗塞に関する論文が散見されるようになり [4]、また感染性心内膜炎の患者では 25%程度に神経合併症を続発すると報告されている[5]。

一方、麻痺や意識障害を主訴として脳神経内科にコンサルトされる患者の一部に、感染症による DIC と脳梗塞を併せて発症している症例があるが、その頻度や予後に関しての検討は極めて少ない。

今回我々は感染症性 DIC に脳梗塞を合併した患者の背景や予後について検討を行い、脳梗塞を生じなかった症例との間に差があるか検討した。

方法

当院は 453 床を有する市中の基幹病院である。2011 年から 2013 年の 3 年間に我々の病院へ入院した患者のうち、DIC の診断名がついた患者を抽出した。その中から基礎疾患が感染症と確定しているものを選択し、悪性腫瘍を有する患者、基礎疾患の感染症が特定できないものは除外された。さらに日本救急医学会と日本血栓止血学会が作成した急性期 DIC スコア[6]を用いて, スコアが 4 点以上のものを DIC と診断された。DIC スコアが 3 点以下の症例は除外された。DIC の診断時に CT もしくは MRI を施行されていた患者が最終的な対象となり、これらの患者についでは敗血症の診断基準である SOFA スコアを用い[7]、スコア 2 点以上の上昇により敗血症と診断されることを確認した。

選択された敗血症性 DIC 患者のなかで発症時のバイタルサイン(血圧、体温、脈拍数、呼吸数)、意識障害の有無、血液検査所見(血算、生化学、凝固系検査)を後方視的に調査した。脳梗塞の診断は CT での低吸収域の出現または MR 拡散強調画像の高信号の出現で行った。これらのデータを用いて、脳梗塞を発症した群と非発症群の間に差があるか検討した。

統計解析

統計パッケージには EzR[8]および Excel を用いた。脳梗塞を生じた群と生じていない群の性別、敗血症の有無や生存率を検討するためにはまず脳梗塞の有無で 2 群に分け、さらに性別、敗血症の有無および生存・死亡でそれぞれ症例数を計上し χ^2 検定を用い検定した。年齢や血液検査データやバイタルサインの比較にはそれぞれの項目の数値について脳梗塞を発症した 8 例と発症していない 19 例の 2 群に分けて Wilcoxon の順位和検定を用い検討した。いずれの項目においても $p<0.05$ を統計学的に有意差ありとした。

倫理的配慮

本研究は藤田医科大学倫理委員会の承認を得て行われた(CI18-273)。患者のプライバシー保護には最大限の注意を払った。後方視的研究であり患者数も多くインフォームド･コンセントは不可能であるためオプトアウトを行った。

結果

a.検討症例の選択

2011～2013 年の間に当院へ入院した患者数は 25017 であり、そのうち播種性血管内凝固と病名が登録された症例は 118 例(0.47%)であった。このうち悪性腫瘍を基礎疾患に生じた DIC 患者は 21 例であり, 感染症以外の疾患で DIC を来した症例を除外すると 97 例であった。次に急性期 DIC スコアが 3 点以下の 32 例が除外され，DIC と診断できた患者は 65 例であった。このうち 51 例が DIC と診断された時点で意識障害を認め､頭部 CT もしくは MRI が DIC を発症し意識障害が出現した後に施行されていたのは 27 例であった(Fig.1)。この 27 例全てが敗血症の診断基準を満たしていることを SOFA スコアを用いて確認した。頭部画像検査が施行された 27 例のうち脳梗塞の発症が確認されたのは 8 例(29.6%)であった。

b.画像診断が施行された 27 例の検討

頭部画像検査を施行された 27 例を脳梗塞発症群 8 例と、非発症群 19 例の 2 群に分けて比較検討を行った。

年齢等の背景と基礎疾患を Table1 に示す。脳梗塞発症群の平均年齢は 84.87±9.16 歳であり、非発症群では平均 76.10±16.18 歳で両者に有意差は認められなかった(p=0.10)。男性 14 例、女性 13 例であり、それぞれ 4 例ずつに脳梗塞が認められ、脳梗塞発症の有無では男女差は認められなかった(それぞれ p=0.90)。27 例のうち生存したのは 9 例(33.3%)であった。生存率については発症群と非発症群を比較してどちらも生命予後は不良であったが、有意差は認められなかった(p=0.21)。また脳梗塞発症群で生存した 2 例についても重度の意識障害が残存しており、機能予後も不良であった。

次に脳梗塞発症群と非発症群でバイタルサインと血液検査所見の比較を行った(Table2)。バイタルサインにおいて全体では収縮期血圧、拡張期血圧のどちらも脳梗塞発症群の方が有意に高値であった(p<0.001)(Fig.2)。脈拍数は脳梗塞発症群の方が少ない傾向があったが有意差がみられなかった(p=0.11)。また脳梗塞発症群は体温の上昇がみられずすべての症例が 37.5℃以下であり、非発症群ではこれより高値であることが多く有意差が認められた(p=0.011)(Fig.3)。

血液検査所見では FDP は脳梗塞発症群の 8 例すべてで急性期 DIC 基準において 3 点となる 25μg/mL より大きい値であったが、非発症群と比較し有意差がみられなかった(p=0.99)。血小板数については脳梗塞発症群では平均値 93000/μL、非発症群では 79000/μL であったが、こちらも有意差はみられなかった(p=0.96)。このほか炎症反応の指標として CRP は脳梗塞発症群で平均値 16.70mg/dL、非発症群で 12.53mg/dL であり、発症群で高値である傾向がみられたが有意差は認めなかった(p=0.16)。PT-INR は脳梗塞発症群に比較して非発症群がやや延長しているが、こちらも有意差が認められなかった(p=0.08)。

c.脳梗塞発症例の画像所見

脳梗塞を生じた患者 8 例の病変の性状について検討した(Table3)。脳梗塞の単発は 2 例であり、6 例は多発性で径が 1.5cm 以上の massive 2 例 (Fig.4)、1.5cm 以下の small のみ 4 例(Fig.5)、両者の混合は 2 例にみられた(Fig.6)。8 例のうち 6 例は診断時の GCS が 6 点以下であり、著明な意識障害を呈していた。脳梗塞症例 8 例において、6 例で椎骨脳底動脈系にも脳梗塞がみられ、内頚動脈系(中大脳動脈領域など)には脳梗塞を認めない例も 1 例存在した。多発性病変が認められた 6 例は頭部 MRA で主幹動脈の閉塞は認めなかった。

考察

DIC の本態は血栓と炎症反応が連動して起きることであり、線溶亢進型と線溶抑制型の 2 種類に分けられ、炎症を起因とした DIC では後者の型を取りやすく必然的に虚血性病変を生じやすい。生体が侵襲を受けると上皮細胞の内側に存在する樹状細胞やマクロファージによって病原体の諸分子(PAMPs<Pathogen-Associated Molecular Patterns>と総称され、エンドトキシンなどが挙げられる)として認識され、その結果サイトカインや HMGB1(High mobility group box-1 protein)などの物質が発生する。HMGB1 とサイトカイン類は組織修復を誘導するが、PAMPs が過剰であったり、全身に拡散すると DIC の原因となる。Hatada らはこの炎症に関連する物質である HMGB1 とトロンビンが同時に血管内に存在すると DIC を引き起こすことを証明した [9]。また、敗血症においては炎症性サイトカインによりトロンボモジュリンや t-PA の産生が低下し、線溶抑制の状態となる [10] ため血栓形成の原因となる。これらの反応に加えて、元々脳組織内ではトロンボプラスチンが多い上にトロンボモジュリンは少ないため、塞栓症を起こしやすい状態にあるという論文もある [11]。一方、脳にはトロンボモジュリンが認められるが脳梗塞の好発部位である橋や被殻には分布が少なく、トロンボモジュリンが脳内においても血栓の形成を抑制するように働いているという考え方もある [12]。敗血症性 DIC は線溶抑制型であるとされるが, その場合プラスミノゲンアクチベーターインヒビター-1 (PAI-1) が高値例では, 循環障害に起因する臓器障害を来しやすく予後不良であるとされる [13]。

本研究において DIC の経過中に測定された検査項目は症例ごとにばらつきがあった。上述したように線溶抑制型 DIC において臓器症状と関連したマーカーには PAI-1、アンチトロンビンⅢ(AT-Ⅲ)、プロテイン C、HMGB-1、白血球エラスターゼ分画フィブリン分解産物(e-XDP)、可溶性フィブリン(SF)などがあるが測定された症例は少なかった。今回の解析で DIC の病勢の指標となる血小板数の減少や FDP の上昇、また CRP などの一般的な検査項目では脳梗塞発症群と非発症群では有意差がみられないことが判明した。今後脳梗塞発症を予見するその他のマーカーの検索が必要である。将来さらに広い検査項目について多数例の集積を行い、さらに前方視的研究が必要である。

敗血症性 DIC に併発した脳梗塞症例 8 例中 6 例で椎骨脳底動脈系にも脳梗塞がみられ、逆に内頚動脈系には脳梗塞を認めない例も存在した。膠原病(全身性エリテマトーデス)においては血管炎の存在が脳梗塞の発症に関与しているとされ、椎骨脳底動脈系の病変が多くの症例で見られたという報告がある [14]。このことから、感染症性 DIC の脳梗塞の発症機序が血管内を浮遊する微小血栓のみではなく、血管炎や血管内の炎症反応が大きく関与している可能性が考えられる。また内

頸動脈系に通常のアテローム血栓性脳梗塞と鑑別の付かない梗塞を生じた症例も存在し(Figure 4)、動脈硬化性疾患は DIC をきっかけに脳梗塞を生じる危険因子であることも否定できない。

起因疾患との関係としては、8 例のうち 5 例は肺炎であった(Table3)。通常大循環経由の微小塞栓は肺で entrap され脳には到達しない。脳への微小塞栓ないしは大塞栓の原因は肺循環にあり、炎症の主体となっている場所である肺循環で発生した微小栓子は脳に塞栓症を生じる可能性は高い。しかしわれわれの検討では肺炎で脳梗塞をきたしやすいという仮説は証明できなかった。

脳梗塞発症群 8 例においては収縮期および拡張期血圧が有意に高値であり、また徐脈となる傾向にあった。脳梗塞や脳出血などを発症したことにより頭蓋内圧が亢進すると交感神経刺激により血圧が上昇し徐脈となる。これは Cushing 現象として知られている[15]。重篤な感染症や敗血症の患者はショックにより血圧が低下する傾向にあり、SOFA スコアや敗血症の存在を予測するために用いる qSOFA スコアにも収縮期血圧および平均動脈圧の低下が診断項目として挙げられている[7]が、脳梗塞発症群においては有意に血圧が上昇しており、Cushing 現象は敗血症性 DIC 患者においても生じると考えられる。敗血症性 DIC 患者がさらに血圧が正常ないしは高値であった場合には脳梗塞を発症している可能性があることを考慮する必要がある。

脳梗塞発症群において体温が上昇しない点は機序が明らかではないが、視床下部のオレキシン陽性細胞の障害によりストレス下においても体温が上昇しなくなるという報告があり[16]、椎骨脳底動脈系の血管の炎症や微小塞栓による視床下部におけるオレキシン陽性細胞の障害が関与している可能性がある。

本研究において DIC 65 例のうち 51 例に意識障害を認めたが、この中で頭部画像検査を施行されたのは 27 例(52.9%)と低率であった。実際に主治医によって頭部画像検査を施行された 27 例の中で 8 例(29.6%)と高率に脳梗塞が確認されているため、他の患者でも頭部画像検査が施行されていれば脳梗塞の発症が確認できた可能性がある。古くから DIC は様々な要因で脳症をもたらしそのため意識障害を生じるという考え方がある[17]。この論文で DIC 脳症は頭部 MR で観察される、脳梗塞や白質病変、PRES(posterior reversible encephalopathy syndrome)を含むとされている。DIC を治療するのは救急医などの神経疾患を専門とする科ではない可能性があり、脳保護薬の投与や脳浮腫の対策を行うか否かを決定するために DIC 患者において意識レベル低下時には頭部 CT や MRI 検査を必ず施行するべきである。また今回対象となった敗血症性 DIC に脳梗塞を生じた患者は半数以上が脳神経内科へ入院しているが(Table3)、DIC の治療においては原疾患の治療が重要であるため、神経症状が先行して脳神経内科での治療がされていれば、原因となった臓器感染症の専門医と連携を取り総合的な治療を行う必要がある。

DIC の治療において、抗凝固療法としてアンチトロンビン活性が低下している場合は AT-Ⅲ製剤を投与することが推奨されている[18]。感染症に合併した DIC においては多くの例で AT-Ⅲ活性が低下するため、AT 製剤の投与が必要となることが多い。海外では DIC の概念が浸透していないこともあり有効性が証明できなかったが[19]、わが国では 2009 年頃から DIC 症例に対して臨床試験を行ったところ有意に DIC からの離脱率が改善することが報告されている[20]。また日本においてはトロンボモジュリン製剤が DIC の治療に使用することが可能であるが、日本版敗血症診療ガイドライン 2016 においては採択されなかった。しかし現在でも研究が続いており一部の患者で有効であるとも報告されている[21]。また血液浄化療法によりサイトカイン吸着を行うことが敗

血症の治療に有効であることも報告されており[22]、これらの治療介入によって敗血症性 DIC からの早期の離脱がなされれば脳梗塞の発症を抑制することにつながる可能性があり、今後の課題である。

利益相反

本研究の共著者は、所属医療機関を通じて武田薬品、塩野義製薬、大塚製薬、ファイザー、帝人、第一三共より助成を受け、また大塚製薬および第一三共からの講義の謝礼を受けた。

参考文献

1. Taylor FB, Jr., Toh CH, Hoots WK, Wada H, Levi M. Towards definition, clinical and laboratory criteria, and a scoring system for disseminated intravascular coagulation. Thromb Haemost 2001; 86: 1327-30.

2. Matsubayashi J, Wakasa T, Yanai H. Thrombosis／embolism and organ dysfunction. Jpn J Diagn Pathol 2018; 35: 264-79.(in japanese)

3. Trousseau A. Phlegmasia alba dolens. Clin Med Hotel Dieu De Paris 1865; 3: 94.

4. Syrjanen J. Infection as a risk factor for cerebral infarction. Eur Heart J 1993; 14 Suppl K: 17-9.

5. Sotero FD, Rosario M, Fonseca AC, Ferro JM. Neurological Complications of Infective Endocarditis. Curr Neurol Neurosci Rep 2019; 19: 23.

6. Gando S, Iba T, Eguchi Y, et al. A multicenter, prospective validation of disseminated intravascular coagulation diagnostic criteria for critically ill patients: comparing current criteria. Crit Care Med 2006; 34: 625-31.

7. Seymour CW, Liu VX, Iwashyna TJ, Brunkhorst FM, Rea TD, Scherag A, Rubenfeld G, Kahn JM, Shankar-Hari M, Singer M, Deutschman CS, Escobar GJ, Angus DC. Assessment of Clinical Criteria for Sepsis: For the Third International Consensus Definitions for Sepsis and Septic Shock (Sepsis-3). Jama 2016; 315: 762-74.

8. Kanda Y. Investigation of the freely available easy-to-use software 'EZR' for medical statistics. Bone Marrow Transplant 2013; 48: 452-8.

9. Hatada T, Wada H, Nobori T, Okabayashi K, Maruyama K, Abe Y, Uemoto S, Yamada S, Maruyama I. Plasma concentrations and importance of High Mobility Group Box protein in the prognosis of organ failure in patients with disseminated intravascular coagulation. Thromb Haemost 2005; 94: 975-9.

10. Levi M, van der Poll T, ten Cate H, van Deventer SJ. The cytokine-mediated imbalance between coagulant and anticoagulant mechanisms in sepsis and endotoxaemia. Eur J Clin Invest 1997; 27: 3-9.

11. Uchiyama S. [Paraneoplastic neurological syndromes: Trousseau syndrome]. Nihon Naika Gakkai Zasshi 2008; 97: 1805-8.(in japanese)

12. Wong VL, Hofman FM, Ishii H, Fisher M. Regional distribution of thrombomodulin in

human brain. Brain Res 1991; 556: 1-5.

13. Morishita E. Other molecular markers related the DIC diagnosis and their significances. Journal of Clinical Laboratory Medicine 2018; 62: 1032-9.(in japanese)

14. Ioannidis S, Mavridis M, Mitsias PD. Ischemic stroke as initial manifestation of systemic lupus erythematosus: A case report and review of the literature. eNeurologicalSci 2018; 13: 26-30.

15. Fodstad H, Kelly PJ, Buchfelder M. History of the cushing reflex. Neurosurgery 2006; 59: 1132-7; discussion 7.

16. Zhang W, Sunanaga J, Takahashi Y, Mori T, Sakurai T, Kanmura Y, Kuwaki T. Orexin neurons are indispensable for stress-induced thermogenesis in mice. J Physiol 2010; 588: 4117-29.

17. Siami S, Annane D, Sharshar T. The encephalopathy in sepsis. Crit Care Clin 2008; 24: 67-82, viii.

18. Kienast J, Juers M, Wiedermann CJ, Hoffmann JN, Ostermann H, Strauss R, Keinecke HO, Warren BL, Opal SM. Treatment effects of high-dose antithrombin without concomitant heparin in patients with severe sepsis with or without disseminated intravascular coagulation. J Thromb Haemost 2006; 4: 90-7.

19. Warren BL, Eid A, Singer P, et al. Caring for the critically ill patient. High-dose antithrombin III in severe sepsis: a randomized controlled trial. Jama 2001; 286: 1869-78.

20. Gando S, Saitoh D, Ishikura H, et al. A randomized, controlled, multicenter trial of the effects of antithrombin on disseminated intravascular coagulation in patients with sepsis. Crit Care 2013; 17: R297.

21. Ito T, Thachil J, Asakura H, Levy JH, Iba T. Thrombomodulin in disseminated intravascular coagulation and other critical conditions-a multi-faceted anticoagulant protein with therapeutic potential. Crit Care 2019; 23: 280.

22. Hirasawa H, Oda S, Nakamura M, Watanabe E, Shiga H, Matsuda K. Continuous hemodiafiltration with a cytokine-adsorbing hemofilter for sepsis. Blood Purif 2012; 34: 164-70.

Figure1 除外診断のアルゴリズムを示す。

Figure2 脳梗塞発症群と非発症群の血圧を比較した結果、収縮期・拡張期血圧ともに脳梗塞発症群は非発症群と比較して有意に上昇を認めた。

Figure3 脳梗塞発症群と非発症群の体温を比較した結果、脳梗塞発症群は非発症群と比較して体温が低下していた。

Figure4 症例 8： 89 歳男性。 脳梗塞は単一病変で粗大(15mm 以上: massive)であった。
a 頭部 MR DWI では右の中大脳動脈領域前半の脳梗塞を認めた。

b MRA では中大脳動脈の分枝の閉塞の可能性が考えられた。
c 胸部 CT では肺炎像を認めた。

Figure5 症例 4： 86 歳男性。著明な意識障害にて救急搬送。小病変の多発症例。
a MRI では右橋中部, 左後頭部に 1.5cm 未満の急性期脳梗塞を認めた。
b MRA には明らかな異常を認めなかった。

Figure6 症例 1： 72 歳男性。歩行困難で救急搬送。 粗大病変(massive)+小病変の混合症例である。
a 頭部 MRI では 15mm 以上の病変+小梗塞の多発を認めた。
b MRA では主幹動脈の途絶は認められなかった。
c 胸部レントゲン写真で肺炎像が認められた。
